# Healthy Hearts for an Abundant Life: Feasibility of a Culturally Adapted Cardiovascular Disease Prevention Curriculum for African American Women

**DOI:** 10.1089/heq.2021.0005

**Published:** 2021-06-10

**Authors:** Reem F. Alsukait, Sara C. Folta, Kenneth Chui, Rebecca A. Seguin, Christine G. Sinclair, Linda B. Hudson

**Affiliations:** ^1^ Community Health Department, King Saud University, Riyadh, Saudi Arabia.; ^2^ Friedman School of Nutrition Science and Policy, Tufts University, Boston, Massachusetts, USA.; ^3^ Public Health and Community Medicine, Tufts University School of Medicine, Boston, Massachusetts, USA.; ^4^ College of Agriculture and Life Sciences, Texas A&M University, College Station, Texas, USA.

**Keywords:** African American, cardiovascular health, community based intervention

## Abstract

**Background:** This study tested the feasibility of implementing Healthy Hearts for an Abundant Life (*HHAL*), a cultural adaptation for African American (AA) women of the evidence-based cardiovascular disease prevention program Strong Women-Healthy Hearts (*SWHH*).

**Methods:** Using a quasi-experimental pre-post study design, this 12-week program was implemented in four faith-based organizations between 2017 and 2018. Eligible participants were AA women between 40 and 65 years who had a body mass index of 25 or higher and were currently sedentary. *HHAL* program participants met weekly for 2-h sessions led by program leaders. The curriculum has four modules: total health; relationships, family, and networks; material security and the environment; and emotional wellness. Each class included walking for 30 min, goal-setting session, and a group dialog called “making it work” for building collective efficacy.

**Results:** Of the 27 participants (mean age=54.2±5.9), 24 completed postassessments (93% retention rate). All outcome measures proved feasible and weekly program attendance was 73%. Findings from in-depth interviews show high satisfaction with the program and suggest extending the class time and adding cooking demonstrations.

**Conclusions:** The culturally adapted *HHAL* proved feasible and was positively received by the participants. Future studies will evaluate the effectiveness of the program.

## Introduction

Cardiovascular disease (CVD) morbidity and mortality demonstrate pronounced disparities between African American (AA) and White women. The prevalence of CVD among AA women is 57% compared to 43% for White women.^1^ Furthermore, AA women have a higher prevalence of some CVD risk factors such as overweight and obesity (82% compared to 64% among White women) and high blood pressure (56% compared to 41% among White women).^1^ Behavioral modifications can reduce the risk of CVD^2^; however, fewer AA women compared to White women meet physical activity guidelines or dietary guidelines.^3^

Strong Women-Healthy Hearts (SWHH) is a 12-week community-based program that has previously demonstrated both effectiveness in decreasing body weight and waist circumference and success at reaching women across the United States.^4,5^ This program was designed for women aged 40 years and older, a group for whom targeted interventions are lacking even though behavioral modifications can reduce risk at any age, including older adults.^6^ In the national dissemination study,^5^ AA women enrolled at rates proportional to their representation in the counties where the program was run; however, they withdrew at twice the rate as White women (36% vs. 17%), and if they completed the program, they did not realize the significant benefits experienced by their counterparts.^5^ Together, these data suggest that program leaders were reasonably successful at outreach to AA women, as evidenced by the interest and response. However, once in the program, AA women's needs were not met, as evidenced by their withdrawal rates.

The relatively few CVD-related interventions developed specifically for AA women indicate that it is possible to achieve significant improvements in risk behaviors and biological parameters when culture, values, beliefs, and unique barriers are considered.^7,8^

Based on this evidence and anecdotal reports from program leaders, we hypothesized that a culturally adapted version of the SWHH program might better meet the needs of AA women. We conducted formative research using the African American Collaborative Obesity Research Network (AACORN) paradigm,^9^ which provides a framework for understanding “deep structure” factors—psychological, cultural, social, historical, and environmental—considered to be critical for maximum salience and effectiveness in cultural adaptations of health programs.^7^

In the formative work, we assessed perspectives of AA women regarding CVD prevention barriers and facilitators, and these insights were used to develop the adapted curriculum entitled Healthy Hearts for Abundant Life (HHAL).^10^ Briefly, participants shared the importance of social support in providing comradery, accountability, and motivation. Structural barriers and sources of stress included racism and a lack of access to healthy food to feed a family. They discussed strategies such as goal-setting and self-care.

The HHAL adapted curriculum incorporated themes from our formative work using the AACORN expanded knowledge domains. These included stress-reducing activities, tools for self-care, metaphorical language based in spirituality, acknowledgement of the role of racism, and strategies and tools consistent with available resources.^9^ These shaped the framing of the core content on diet, physical activity, and heart disease prevention from the original SWHH curriculum.

To help address gaps in the nutrition intervention literature for AA women, we conducted a pilot study to determine the feasibility of implementing the HHAL program and collecting outcome measurements and to assess whether the adaptation would be a cultural fit. Another goal was to calculate effect sizes and intraclass correlation coefficients (ICCs), which are necessary to design a fully powered randomized controlled trial.

## Methods

This pilot was designed as a quasi-experimental pre-post study. The 12-week program was implemented between November 2017 and April 2018 in four predominately AA churches in the northeastern United States. The program was conducted in churches because 62% of AA women report an affiliation with an historically Black church, with most attending at least weekly.^11^ Furthermore, the study team had pre-existing relationships with several predominantly AA churches and utilized these connections to recruit churches. Each church received $500 for hosting the program. The two program leaders were community-based nutrition professionals recruited through a professional organization designed for nutritionists and dietitians of color.

To be eligible to teach HHAL, the program leaders attended a 5-h training workshop. They received $25 per hour for administering the program. The Tufts University Social, Behavioral, and Educational Research Institutional Review Board approved all study procedures.

### Intervention

Both SWHH and HHAL class topics have been included (Appendices A1 and A2). HHAL group members met for 2 h every week and were led by the trained program leaders. The HHAL curriculum had four modules: total health; relationships, family, and networks; material security and the environment; and emotional wellness. Each class included six components: (1) an introduction to the session's topic, (2) a leader-led discussion for knowledge dissemination, (3) walking for ∼30 min, (4) a group dialog called “making it work” to build collective efficacy and to discuss common barriers, (5) a journaling and goal-setting session to help participants integrate behaviors into their everyday lives, and (6) a wrap-up. While the intervention included some metaphorical language based in spirituality, it was fundamentally secular in nature. A recipe booklet with culturally appropriate recipes was developed for the program.

### Impressions of cultural fit

Participants and program leaders were interviewed after program completion using semi-structured interview guides to better understand the resonance of the program within AA culture. The key topic areas for the interviews were (1) overall experience, program curriculum, and structure; (2) curriculum modules and understandability of materials and handouts; and (3) perceptions of participants' own abilities to make the suggested behavior changes given the resources available in their environment. Three participants from each group were randomly selected and invited to participate in a phone interview administered by research assistants. Participants gave verbal consent at the beginning of the call to be interviewed and recorded, and all interviews were transcribed. The codebook used for qualitative data analysis was initially based on the interview guide; it was then refined in an iterative process based on the initial interviews. Standard qualitative methods were used.^12^ Two coders worked on the analysis, and intercoder-reliability was established at 80% agreement or greater. NVivo Pro 11 software (QSR International, Doncaster, VIC, Australia) was used during analysis.^13^

### Outcome measures

**FIG. 1.**
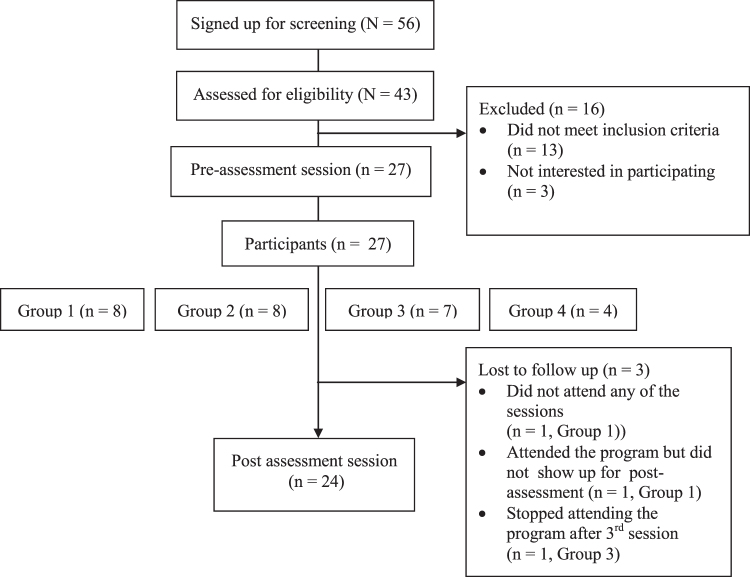
Flowchart of participant selection for HHAL study, 2017–2018. HHAL, Healthy Hearts for an Abundant Life.

In collaboration with the host churches, participants were recruited through posting promotional flyers on-site, using the churches' email lists, and conducting informational on-site visits where research personnel provided sign-up sheets for interested church attendees. Research assistants called potential participants from the sign-up sheets to assess their eligibility. The inclusion criteria were women aged 40–65 years who self-identified as AA, spoke English, had a body mass index (BMI) of 25 or higher, were currently sedentary and not meeting the Physical Activity Guidelines for Americans,^14^ and could safely engage in moderate physical activity according to the Physical Activity Readiness Questionnaire (PAR-Q).^15^ Participants were excluded if they failed to provide informed consent, were regularly participating in any other lifestyle modification program, were pregnant, or were planning to move outside the area within 5 months. Women deemed eligible during the telephone screening were invited to a preintervention assessment session. Informed consent was obtained during this session before assessment. The study's participant flow is depicted in Figure 1. Each participant received a $75 gift card at each assessment point (both pre- and postintervention).

All measurements were taken at the preintervention assessment session, which occurred within 2 weeks of the intervention start, and again at the postintervention assessment session, which occurred within 2 weeks after the final class.

Demographic information included age, marital status, occupational status, education, and income level.^16^ Trained research assistants performed anthropometric measurements in triplicate.^17^ These measurements comprised body weight to the nearest 0.5 kg and height to the nearest 0.5 cm, and both were used to calculate BMI. Body fat percentage was assessed using bioelectric impedance (Tanita DC-430 MAP), and blood pressure was measured to the nearest 5 mmHg using an automated monitor.^18,19^ Dietary intakes were measured by three 24-h recalls (2 weekdays and 1 weekend day) using the ASA24^®^ dietary assessment tool.^20^ Participants received text messages from the study team on randomly assigned days that reminded them to log into ASA24 and report their intake. Physical activity was assessed by administering the International Physical Activity Questionnaire (IPAQ) long form, which has been validated in multiethnic populations.^21^ Cardiorespiratory fitness, which is strongly associated with decreased CVD risk, was measured using the Siconolfi step test since this method has been validated for the estimation of VO2 max in very-low-fitness populations.^22^ Participants were asked to step up and down on a 10-inch bench, and their heart rates were monitored and recorded at the end of each of the test's three phases. The ICC was estimated for body weight, systolic blood pressure, and diastolic blood pressure using cluster-adjusted paired *t*-tests.

Psychosocial factors were assessed using validated instruments that were compiled and administered via an online survey during the pre- and postintervention assessments. To assess self-efficacy for eating and exercise behaviors, both the 20-item Weight Efficacy Lifestyle Scale and the 5-item Exercise Self-Efficacy Scale were used.^23,24^ Perceived social support was assessed using the 23-item Sallis Social Support Scales for Eating and Exercise Behavior.^25^

Descriptive statistics of the demographic information and baseline values were compiled and tabulated. Changes in body weight, dietary intake, physical activity duration and frequency, blood pressure, cardiorespiratory fitness, and psychosocial factors were assessed using paired t-tests or Wilcoxon signed-rank tests for participants with both timepoints (pre-post). All statistical analyses were performed in SAS 9.3 (SAS Institute, Cary, NC). As per the goal of acquiring information to design a randomized, controlled trial, the ICC for body weight was determined, which will be the main outcome in future trials.

### Process evaluation

Program leaders were asked to complete weekly online questionnaires about their class teaching experience and document any challenges they faced as a measure of dose and fidelity to the program. Program participants were asked to complete a midpoint online assessment at the 6-week mark to assess any changes in health status (such as recent injuries or illnesses) and program satisfaction. Adherence was operationalized as the number of sessions attended by each participant, as measured with the class attendance sheets completed by the program leaders. Retention was defined as the number of participants who completed postassessments. Reach into the target population was estimated from the number of people who signed up to be screened yet did not participate in or were not eligible for the study.

## Results

Baseline data were obtained from 27 study participants. The mean age of the sample was 54.2 years (with ages ranging from 40 to 61 years); 64% had a bachelor's degree or above, 44% had an income of $75,000 or more, and 68% were employed full time (Table 1).

**Table 1.** Demographic Characteristics of Study Participants at Baseline (*n*=27)

	Mean (SD)
Age (*n*=27)	54.2 (5.9)
Education (*n*=25)	*n* (%)
Bachelor's degree or above	16 (64)
Associate degree	4 (16)
High school diploma/GED or less	5 (20)
Relationship status (*n*=25)	
Living with a partner (married or unmarried)	8 (32)
Living without a partner	17 (68)
Household income (*n*=25)	
$75,000 or more	11 (44)
$25,000 to $74,999	9 (36)
Less than $25,000	3 (12)
Prefer not to reply	2 (8)
Work outside of home (*n*=25)	
Yes, full time	17 (68)
Yes, part time	4 (16)
No	4 (16)

GED, General Educational Development; SD, standard deviation.

### Impressions of cultural fit

The two program leaders and 10 participants took part in the telephone interviews. Overall, the program leaders and participants had a positive experience with the program. Several themes emerged (Table 2). The program leaders found the HHAL curriculum comprehensive, and the topics covered were relevant and important to the women in their groups. One program leader noted how the class on racism and emotional wellness generated in-depth discussions among participants. The program leaders also recommended adding 30 min to the class time because of the difficulties fitting everything within the allotted 2 h.

**Table 2.** Qualitative Interviews to Gauge Cultural Fit of Healthy Hearts for an Abundant Life

Domain	Themes	Representative Quotes
Social support and comradery	The group setting improved accountability and motivation for participants	What I liked the most was meeting other women of color and doing this experience together. I got to make some friendships and share issues in life as women of color (Program Participant Group 4 Participant 2) I think accountability and just being able to meet with people who had the same goal of living an abundant healthy life with no judgment, but encouragement was big. The information and everything were good, and exercise was good, and cooking was good but the really the best part was having a group of women who were looking to do the same thing without judgment. (Program Participant Group 3 Participant 2) I mean we would text each other you know to see if people were coming. If the person wouldn't come, we would text them and make sure they were okay, or I mean we had a thread together on the phone, so we were really close. (Program Participant Group 4 Participant 2)
Racism and stress	Racism class fitted with the overall program and participants' lived experiences	The class on racism was absolutely relevant. Unfortunately, it is a part of their reality. It affects a lot of stuff because with more stress, you do more emotional eating and don't care as much about being healthy and physically active. Not having this class would be a disservice to the program. (Program Leader 1)
Community	Examples of linking the class material to the community	We had to go into our community and look at different marketing materials and how it targets people of color and what does that mean for us and what we can do to help other people understand how [we] were being targeted, you know? (Program Participant Group 4 Participant 2) You know, there are liquor stores and fast food restaurants on every corner in the hood, but in the suburbs, you don't see that. And it made me aware of that and I'm a teacher, and you know I hear my students say 3 nights a week that they're at McDonalds I'm like “Dude, what”? But, that's what's there, and that's what convenient for those families. So it made me really aware of and sensitive to the fact that that's what there. (Program Participant Group 1 Participant 3)
Emotional wellness and spirituality	The role of faith in living a healthy life was paramount	God and prayer is an important role in helping us to sort of achieve some of the goals that we were trying to accomplish so um, I think it was definitely a good fit. You know, like we prayed, so it was definitely a good fit with my own personal beliefs. (Program Participant Group 2 Participant 1) I'm a woman of faith and having my faith in God and going to church and being involved in church is very important to my heart health and my health period. You know being with my family keeps me healthy, being with friends keeps me healthy setting limits is keeping me healthy and that is an Abundant Life. (Program Participant Group 3 Participant 2)
Relationships and family role	Participants discussed the struggles of balancing work and family	We are so busy, but we need to take time for ourselves for self-care. We do so much for our families, but you know we can't be as effective if we're not healthy. So, I guess continue to drill that we need to take care of ourselves and be as healthy as possible (Program Participant Group 2 Participant 3).
Cultural resonance	Having a women of color as a program leader and using spices from the recipe book enabled behavioral changes	Just having someone, a nutritionist, that deals with women of color, you know older women of color, not even of color, just women period. When I get in class, I'm really taking time to do this, I really want to hear about women, I don't want to hear about anything else, I want to hear nutrition, women, exercise. (Program Participant Group 1 Participant 5) you know, culturally we operate this way, but then individually within that culture we operate differently as well. I know people think that people are monolithic we aren't- we aren't all the same. (Program Participant Group 3 Participant 2) I believe it fits my lifestyle because I'm trying to trying to be healthy and eat better and exercise and go to the gym and keep my calorie count, you know, at a healthy place, and my blood pressure, and I've been really, really consistent, I've actually been able to come off of blood pressure medicine since I started. (Program Participant Group 2 Participant 3) I love the recipe thing. It uses spices. I think that's a real cultural thing because I think in this area of Massachusetts food does not have a lot of seasoning in it. But what I liked about this one is it used a lot of different spices and seasoning and put combinations of food, like fruit with greens. (Program Participant Group 4 Participant 1)
Challenges with ASA24 dietary recalls	Using the ASA24 dietary recalls was time consuming and frustrating	I can't keep up with it. As much as I wish I could have, I missed some days and it took a lot to log in what you ate because we had to go back and measure sizes and that was a lot of work. I was pretty frustrated trying to figure out how to put all the information in. I think they would need to walk people through how to do that at the first training. (Program Participant Group 1 Participant 1)

*n*=12: Themes and representative quotes.

The program participants appreciated building on shared lived experiences and knowledge. They discussed how the relationships cultivated during the program kept motivation and accountability high. They also described incorporating dietary changes at home and reading food labels more consciously. The participants specifically referenced how the class on built environment heightened their awareness of targeted marketing in AA communities.

### Outcome measures

As shown in Table 3, at baseline across the four churches, the mean BMI was 35.9 kg/m^2^ (standard deviation [SD]: 6.7), and the mean pre-post change was 0.3 kg/m^2^ (95% confidence interval [CI]: −0.9 to 7.2). The mean percent body fat at baseline was 45.6% (SD: 5), and the mean pre-post change was −0.1% (95% CI: −0.8 to 3). Mean systolic blood pressure at baseline was 138.4 mmHg (SD: 18.16), and the mean pre-post change was 4.8 mmHg (95% CI: −0.9 to 7.2); mean diastolic blood pressure was 83.1 mmHg (SD: 7.8), and the mean pre-post change was 0.6 mmHg (95% CI: −0.8 to 3). For cardiorespiratory fitness, the participants' mean VO2 max estimate was 17.8 (SD: 3.4), and the mean pre-post change was 0.9 (95% CI: −2.2 to 0.3). Participants' median self-reported Metabolic Equivalent of task minutes per week of physical activity was 2934 (interquartile range [IQR] 6263), and the mean pre-post change was 610 (IQR 5457). For dietary intake, participants reported a mean intake of ∼2 cups of fruit (SD: 1.3) and vegetables (SD: 1) per day; the mean pre-post changes were −0.5 cups of fruit per day (95% CI: −1.1 to 0.3) and 0.1 cups of vegetables per day (95% CI: −0.6 to 1.1). The ICC for body weight was between 0.025 at baseline and 0.086 at follow-up.

**Table 3.** Study Outcomes at Pre- and Postintervention

	Preintervention Mean (SD)	Postintervention Mean (SD)	Change (95% CI)^a^
Anthropometrics			
Body mass index (kg/m^2^) (*n*=24)	35.9 (6.7)	36.2 (6.4)	0.3 (−0.9 to 7.2)
Body fat (%) (*n*=22)	45.6 (5)	45.5 (5.5)	−0.1 (−0.8 to 3)
Physical activity^b^			
MET minutes/week (*n*=17)	2934 (6263)	3544 (9424)	610 (5457)
Dietary intake			
Mean fruit intake (servings/day) (*n*=13)	2 (1.3)	1.5 (1.4)	−0.5 (−1.1 to 0.3)
Mean vegetable intake (servings/day) (*n*=13)	2.1 (1.0)	2.2 (1.6)	0.1 (−0.6 to 1.1)
Blood pressure			
Systolic blood pressure (mmHg) (*n*=23)	138.4 (18.2)	143.1 (19.3)	4.8 (−0.9 to 7.2)
Diastolic blood pressure (mmHg) (*n*=23)	83.1 (7.8)	83.7 (7.6)	0.6 (−0.8 to 3)
VO2 max (*n*=13)	17.8 (3.4)	18.7 (2.7)	0.9 (−2.2 to 0.3)

^a^ All changes were nonsignificant at *p*=0.05 level.

^b^ Median (IQR).

CI, confidence interval; IQR, interquartile range; MET, metabolic equivalent of task.

Among psychosocial measures, within the Social Support for Diet and Exercise Behavior Scale, there was a significant pre-post decline in participants' ratings of family members complaining or criticizing them for exercising (−0.62, *p*-value=0.029). Friends' discouragement to exercise declined significantly (−2.05, *p*-value=0.01). The pre-post change in all remaining psychosocial and outcome measures were non-significant.

### Process measures

Despite the small stipend, all four churches approached agreed to run the program. There were no issues with space availability or scheduling, which indicated the feasibility of implementation using the available resources in the community. The majority of study participants attended the postassessment (89% retention rate), and weekly class attendance (adherence) was 73%. During the midpoint satisfaction survey, all participants were “very satisfied” or “generally satisfied” with their overall experience in the program (100%), and the majority were “very satisfied” or “generally satisfied” with the progress they made because of their participation in the program (95%). The majority of study participants (83%) rated the program's effectiveness as “excellent” or “very good” in meeting the needs of group members for making lifestyle changes and fostering relationships between group members. The program leaders in their weekly online reflections documented the dose and fidelity of the program delivered. This included how certain class topics created robust conversations among participants that caused them to shorten or postpone some content. Additionally, program leaders highlighted the difficulty of completing both the exercise component and the classroom content within the allotted 2 h, partly due to delays in participant arrival to the classroom and delayed walking sessions. One leader rescheduled the exercise component to occur at the beginning of class to adjust for shortened daylight during winter. Overall, program leaders had to cancel 2–3 classes because of inclement weather, but they were able to combine sessions and meet with their groups on alternative days. Our target enrollment sample was 30 across all sites. Recruitment through churches took ∼1 month and led to 56 participants signing up for screening, 13 of whom were subsequently unreachable (23%) and 16 of whom (28%) were ineligible. We concluded enrollment at 27 participants, close to our goal, for logistical reasons. This indicates there was interest in the program, but that we may need to reconsider aspects of the recruitment process, for example by requiring that multiple forms of contact information are provided.

Using the ASA24 dietary recall tool proved a challenge. Only half of the study participants completed both pre- and postintervention 24-h recalls (13 out of 27). The participants were invited to participate in an additional feedback interview to better understand their challenges via ASA24; four participants gave 20-min interviews and received an additional $25 gift card each. Overall, they expressed frustration with the website, the length and repetition of the questions, and an inability to complete the recalls immediately after receiving the text message reminders (i.e., due to being at work). The Siconolfi step test presented some technical difficulties with the heart rate monitor, which prevented us from reaching the full sample (13 out of 27).

## Discussion

This pilot study demonstrated the feasibility of implementing HHAL in church settings and supports the validity of HHAL, a culturally adapted evidence-based program designed for AA women. The participants' adherence and retention rates were within the range of those observed in similar studies, which supports the program's feasibility. The adherence rate in HHAL was 73%, and six similar group-based community CVD programs for AA women reported adherence rates of 53% to 89%.^26–31^ HHAL experienced a retention rate of 93%; of eight similar studies, only two reported retention rates greater than 90%.^26–33^ While other CVD interventions for AA women focused on diet and physical activity,^8^ HHAL adopted a total health approach and addressed cardiorespiratory health, stress reduction, and the built environment. Additionally, the program used multiple cultural adaptation levels.^34^

The program was delivered by two AA women of similar age to the participants. Each program leader led two of the groups separately (they did not co-teach). The material and recipes were designed to reflect the target population. We implemented the program in church settings to incorporate aspects of the communal identity frame in an attempt to increase the effectiveness of interventions.^8^ Furthermore, the program recognized the role of AA women as caretakers and incorporates self-care tools. Additionally, knowledge sharing was built into the program in recognition of the importance of personal experience to leverage behavior changes among AA women.^34^

The findings from the in-depth qualitative interviews with the program leaders and study participants showed an overall positive program experience. Specifically, the participants highlighted how they appreciated the sense of comradery from the group and being able to share experiences and learn from other AA women. Social relationships and connectedness are known protective factors for CVD risk among AA women.^35^

The core content related to nutrition and physical activity education for HHAL was the same as for SWHH; however, in HHAL, where both deep and surface structural factors were incorporated, the program was better received and resonated more with AA women, as evidenced through their higher attendance and adherence rates. These results are encouraging given the evidence that taking culture, values, and beliefs into account can significantly improve modifiable risk factors among this group.^9^ Recommendations from the qualitative interviews, such as having more support for ASA24 dietary recalls, will be considered in future HHAL iterations.

This study had several strengths. The adaptation was based on a “deep structure” cultural framework and formative research, and the overall study and the intervention were led by AA women. This helped ensure that the adapted curriculum was grounded in lived experiences. The curriculum structure and outcome measures proved feasible. Specifically, the Siconolfi step test captured cardio fitness health and produced reasonable VO2 max estimates, unlike the 1-mile Rockport test. In a previous study with a similar group of midlife Black/AA women, the 1-mile Rockport test's average walk times were too long for an accurate estimation formula.^26^ Although ASA24 is a cost-effective and reliable instrument,^36^ its usability should be tested in the study population. In this study, only half of the participants completed all three recalls. The majority of these recalls occurred 1 or 2 days after the unannounced reminder text messages, leading to reactivity bias in reported diet intake. While three recalls is the gold standard, other studies found more than two recalls burdensome for this population.^37^ Future studies should hire staff, preferably nutrition professionals, to administer the ASA24 to ensure the accuracy of the collected dietary data and to reduce participant burden.^38,39^

This study has several limitations. Several dietary intake data were missing because of technical difficulties with ASA24, as described above. Another potential limitation is that in one of the groups, only one person participated in the interview, potentially biasing responses from that group. Furthermore, the participants were relatively more educated and had a higher income compared to the national average, which might have affected adherence to the program. Finally, all the churches were in a northeastern American city, and participants were compensated for their participation, which might have affected generalizability.

## Conclusions

Overall, this pilot study showed the feasibility and acceptability of *HHAL*, a cultural adaptation of the evidence based program *SWHH*. Culturally adapted programs can lead to significant improvements in diet and weight outcomes among AA women.^40^ Cultural considerations differ across AA women.^8^ *HHAL* specifically targets midlife AA women and used formative work to incorporate their preferences and needs that may differ from other subgroups.^34^ The high hypertension rate observed among our participants further emphasized the importance of addressing CVD risk among AA women. Such programs could potentially reduce CVD morbidity and mortality in this underserved population with pronounced health disparities. Future work will investigate the effectiveness of *HHAL* and demonstrate the link between cultural variables and health outcomes.

## Abbreviations Used

AA: African American
AACORN: African American Collaborative Obesity Research Network
BMI: body mass index
CI: confidence interval
CVD: cardiovascular disease
GED: General Educational Development
HHAL: Healthy Hearts for an Abundant Life
IPAQ: International Physical Activity Questionnaire
IQR: interquartile range
MET: metabolic equivalent of task
PAR-Q: Physical Activity Readiness Questionnaire
SD: standard deviation
SWHH: Strong Women-Healthy Hearts

## Appendix

**Appendix A1.** Healthy Hearts for an Abundant Life: Class Curriculum (Once Per Week)

Week	Module	Class
1	Module 1 : Total health	Class 1: Shaping the class experience; visualizing an Abundant Life; people of color and heart disease; people of color and body image; heart health and total health
2		Class 2: Stress and heart disease; weight control for heart health; self-monitoring for an Abundant Life
3		Class 3: Eating right for an Abundant Life: a heart healthy eating pattern; eating by HEAR
4		Class 4: Eating right for an Abundant Life: breakfast, lunch, and dinner
5		Class 5: Eating right for an Abundant Life: snacks and beverages; lowering sodium and added sugars
6		Class 6: Eating right for an Abundant Life: Menu planning; putting it all together and making it work
7	Module 2: Relationships, families, and networks	Class 7: The importance of self-care and communication for an Abundant Life; how to make the Abundant Life work for those you love
8	Module 3: Material security and the environment	Class 8: Creating an Abundant home for an Abundant Life; harnessing the power of community for an Abundant Life
9		Class 9: The food environment where you live; how to live Abundantly in the built environment around yo
10	Module 4: Emotional wellness	Class 10: Targeted advertising and how it affects the pursuit of an Abundant Life; navigating nutrition advice for an Abundant Life
11		Class 11: Strategies for sustaining Abundance over a lifetime; prioritizing what strategies work for an Abundant Life; how race, culture, and spirituality work in an Abundant Life
12		Class 12: Celebrating our history, culture, and community; continuing to live an Abundant Life that works for us, in our community

**Appendix A2.** Strong Women-Healthy Hearts: Class Curriculum (Twice Per Week)

Week	Class
1	Class 1 : Women and heart disease (Leader-directed discussion)
	Class 2: Weight control for heart health, part 1 (Leader-directed discussion, goal-setting exercise)
2	Class 4: Weight control for heart health, part 2 (Leader-directed discussion, interactive quiz)
	Class 5: Heap on the vegetables and fruits, part 1 (Leader-directed discussion, demonstration)
3	Class 6: Heap on the vegetables and fruits, part 2 (Cooking)
	Class 7: Emphasize the right fats, part 1 (Leader-directed discussion)
4	Class 8: Emphasize the right fats, part 2 (Demonstration)
	Class 9: Accentuate whole grains, part 1 (Leader-directed discussion)
5	Class 10: Accentuate whole grains, part 2 (Tasting, cooking)
	Class 11: Revere low- and nonfat dairy, part 1 (Leader-directed discussion)
6	Class 12: Revere low- and nonfat dairy, part 2 (Cooking)
	Class 13: Target heart-healthy proteins, part 1 (Leader-directed discussion)
7	Class 14: Target heart-healthy proteins, part 2 (Cooking)
	Class 15: Putting it all together, part 1: grocery shopping, breakfast (Leader directed discussion)
8	Class 16: Putting it all together, part 2: lunch; salt (Group discussion, tasting, leader-directed discussion)
	Class 17: Putting it all together, part 3: snacks; nuts; treats (Group discussion, tasting, leader-directed discussion)
9	Class 18: Putting it all together, part 4: dinner; healthy cooking; alcohol (Group discussion, leader-directed discussion)
	Class 19: Putting it all together, part 5: menu planning (Leader-directed discussion, written activity)
10	Class 20: Putting it all together, part 6: menu planning, eating by HEART and weight loss (Leader-directed discussion, written activity)
	Class 21: Weight control for a lifetime, part 1 (Group discussion, leader directed discussion)
11	Class 22: Weight control for a lifetime, part 2 (Leader-directed discussion, role-playing)
	Class 23: Weight control for a lifetime, part 3 (Leader-directed discussion, role-playing, group discussion)
12	Class 24: Stress reduction (Leader-directed discussion, meditation exercise)
	Class 25: Heart Healthy Potluck and Awards Ceremony
